# Protein for Life: Towards a focussed dietary framework for healthy ageing

**DOI:** 10.1111/nbu.12312

**Published:** 2018-02-13

**Authors:** E. J. Stevenson, A. W. Watson, J. M. Brunstrom, B. M. Corfe, M. A. Green, A. M. Johnstone, E. A. Williams

**Affiliations:** ^1^ Human Nutrition Research Centre Institute of Cellular Medicine Newcastle University Newcastle Upon‐Tyne UK; ^2^ School of Experimental Psychology University of Bristol Bristol UK; ^3^ Department of Oncology University of Sheffield Sheffield UK; ^4^ School of Environmental Sciences University of Liverpool Liverpool UK; ^5^ The Rowett Institute University of Aberdeen Aberdeen Scotland

**Keywords:** ageing, health, product development, protein, sarcopaenia

## Abstract

‘Ageing well’ has been highlighted as an important research area by the World Health Organization. In the UK, healthy ageing has been identified as a priority research area by multiple Research Councils and is a key NHS priority. Sarcopaenia, the decline of muscle mass/strength and a key component of healthy ageing, can have a major impact on quality of life and is associated with premature mortality. Increasing protein intake at all stages of the life course may help to reduce the rate of muscle decline and the onset of associated health conditions. However, there is a lack of understanding of the social, demographic and psychological drivers of food choices surrounding protein intake. This report describes the multidisciplinary approach that has been adopted by the *Protein for Life* project to create a framework for the development of palatable, cost‐effective higher‐protein foods suitable for an ageing population.

## Introduction

Ageing is associated with sarcopaenia, the decline of muscle mass and strength with age (Santilli *et al*. 2014). Sarcopaenia is a chronic condition affecting physical functioning and is a key determinant of quality of life and premature mortality (Metter *et al*. 2002). There are many complex underlying causes of sarcopaenia, including a sedentary lifestyle, and poor diet and nutritional status (Santilli *et al*. 2014). It is also recognised that low physical activity is a major driving factor of muscle wastage with age (Steffl *et al*. 2017). The benefits of increased protein intake for older adults to augment muscle mass has led to suggestions that protein intake should be greater than the UK reference nutrient intake (RNI) of 0.75 g of protein per kg of bodyweight per day (Bauer *et al*. 2013; Volpi *et al*. 2013). Adults tend to skew their protein intake towards evening meals, which is an inefficient consumption strategy for daily muscle protein synthesis (Paddon‐Jones *et al*. 2015). The consumption of several smaller portions of protein throughout the day could be a superior strategy (Bauer *et al*. 2013; Farsijani *et al*. 2017). Protein‐rich snack foods might represent an ideal opportunity to increase total protein intake in older adults. However, there are few mainstream snack‐based products designed to meet the particular requirements, preferences and budget of older adults.

Research to improve protein intake has typically focused on older adult populations. However, given the long‐term determinants of sarcopaenia, such as poor nutrition and limited physical activity over a number of years, there needs to be greater focus on adults at earlier points in the life course, with mid‐life regarded as a key point for intervention. While there has been some investigation into protein intake by age group (Davey *et al*. 2007), there is a lack of understanding of the social, demographic and psychological drivers of food choices surrounding protein intake. This is despite a wide range of evidence demonstrating the importance of these factors in determining broader dietary behaviours. Examining how the drivers of, and barriers to, protein intake vary by age will help to tailor interventions to encourage appropriate protein intake at different points of the life course, and generate guidelines that can inform new product development or reformulation to encourage increased protein intake and support healthy ageing. In this context, a multidisciplinary team of researchers working on the *Protein for Life* project will assess the factors related to protein intake of three different life stages: mid‐life (40–54 years), younger old (55–69 years) and older old (70+ years).

The *Protein for Life* project was funded by the Research Councils UK ‘Priming Food Partnerships’ initiative, which is supported by four councils: Biotechnology and Biological Sciences Research Council (BBSRC), Medical Research Council (MRC), Engineering and Physical Sciences Research Council (EPSRC) and Economic and Social Research Council (ESRC). The initiative supports pre‐competitive research, with the aim of stimulating innovative research and technological advances of relevance to the food industry. The project will run for 18 months (July 2017–December 2018) and has five key objectives related to the development and dissemination of guidelines for the formulation of palatable, cost‐effective, higher‐protein foods for an ageing population.

## Objective 1: Develop a multidisciplinary evidence base around protein intake and decision‐making in older adults

There is little information on behaviours influencing protein intake at a population level. The first objective of the project is to develop an evidence base around the protein intake of adults aged 40 years and older. A multidisciplinary approach will be used, encompassing the analysis of large consumer datasets and qualitative assessments of food choice.

### A quantitative assessment of food choice

Analyses will be conducted on three data sources to identify patterns in behaviours related to protein intake by demographic characteristics. The focus will be on identifying protein intake in the UK population (by age), the types of foods that contribute to protein intake, and the social and demographic characteristics associated with purchasing and consumption behaviours. Understanding the types of foods that contribute most to protein intake and why people choose these foods is imperative for constructing a data‐driven evidence base to identify potential products that might offer greater choice to consumers.

The first data source will be the *National Diet and Nutrition Survey* (*NDNS*) (Years 2011–2014), which is a nationally representative survey of dietary intake. The *NDNS* will allow exploration of patterns in overall protein intakes [total intake (g) and as a percentage of total energy intake], as well as the main foods and food groups that contribute to protein intake. These measures will be calculated for each of the target age groups (40–54, 55–69 and 70+ years) and will be stratified by sex and socio‐economic status (SES). Summary statistics for these measures will be reported, and regression models will be used to analyse associations between social and demographic factors and measures of protein intake. Cluster analysis will be used to classify dietary behaviours in relation to protein intake by type of food. The resulting classification will develop a data‐driven typology of the sources of protein for particular groups. The analysis will be undertaken for individuals aged 40–54, 55–69 and 70+ years separately, and regression analysis will be used to explore how the typologies relate to overall protein intake.

Results from the *NDNS* analyses will be considered alongside data on consumer behaviours from loyalty card data, provided by one of the largest UK supermarkets. The novel data will include objective measures of food purchasing behaviours and product nutritional information. Although these data may be less representative of the UK population than the *NDNS*, they provide novel information on objective food purchasing behaviours. Data from a UK food manufacturer from their market research on attitudes to protein has also been acquired, including the acceptability of certain products, which will feed into the product development process.

In a third and complementary approach, protein intake data from two trials with older adults centred in Sheffield *(FIT* and *Nana*) (Forster *et al*. 2012; Timon *et al*. 2015) will be assessed. In total, these studies yielded 300 4‐day food diaries, which offer a detailed and fine‐grained snapshot of the quantity, quality and timing of protein intake in a regional population of older adults. Analyses will be stratified by sex, age group and SES and will focus on the nature (quality and type), quantity and timing of protein intake.

### A qualitative assessment of food choice

A recently published study of 28 older adults (27 females and 1 male) aged between 65 and 93 years (mean 81 years) (Best & Appleton 2013) used thematic analysis to identify the main reasons for consumption and non‐consumption of protein‐rich foods. Three main themes were identified related to product‐based reasons (*e.g*. appearance and taste), environmental‐based reasons (*e.g*. convenience and effort to cook) and cognitive‐based reasons (*e.g*. nutritional knowledge and health beliefs) (Best & Appleton 2013). However, the authors acknowledged that further research is needed to establish how each theme relates to actual consumption of protein‐rich foods. There is also a lack of understanding of the social, demographic and psychological drivers of food choices related to protein intake. Therefore, barriers and facilitators to intake of protein‐rich foods will be investigated in a sample of community‐dwelling healthy mid‐life (40–54 years), young old (55–69 years) and older old adults (70+ years). Focus group discussions will be conducted to explore in more detail the themes generated by Best and Appleton (2013), focusing on psychological and behavioural drivers of food choice. A structured topic guide will be used to facilitate these focus group discussions.

To explore the themes identified in relation to actual protein consumption (as recommended by Best and Appleton), the participants’ usual consumption of protein‐rich foods will be assessed using a food frequency questionnaire (FFQ). Participants will be asked to identify the frequency of consumption of white meat; red meat; processed meats; white fish; oily fish; shellfish; eggs; milk; yogurt, custards and blancmanges; soft and hard cheeses; and plant‐based proteins. To capture the diets of vegetarians and vegans, questions about consumption of seeds; nuts; mushrooms; beans; pulses and protein substitutes will be included.

## Objective 2: To identify design constraints for academic and industry partners

The current literature on the role of dietary proteins for an ageing population for lifelong health will be summarised in a report aimed at industry partners. Current dietary patterns from the 2010 *NDNS* and information from the quantitative assessment of food choice will be considered. The literature will be reviewed on essential amino acids from different protein sources and suggested human amino acid requirements for healthy ageing. The report will also consider popular protein foods and their role in nutrition and health, including meat‐based protein sources, and identification of plant‐based alternatives used in replacement. Environmental sustainability and public health issues related to protein intake and faced by the food industry will also be considered. The report will detail the protein sources currently utilised by industry partners and in which types of products they are found (*e.g*. baked, fresh, liquid).

### Choice architecture

In addition to exploring consumer behaviours and specific attitudes towards increased protein consumption, techniques will be drawn from experimental psychology to develop predictive models of food choice. Behavioural and neuroimaging studies show that choice is mediated both by the pleasure that is anticipated from a meal (governed by the mesolimbic dopamine system and opioid systems) and by ‘top‐down’ inhibitory control, based on long‐term concerns such as effects on bodyweight and health (Hare *et al*. 2009). More recently, it has become clear that food choice is also influenced by ‘expected satiety’, a medium‐term, meal‐to‐meal concern to ensure satiety between meals (Brunstrom *et al*. 2016). To understand and quantify their relative role, psychophysical methods will be used that have been previously developed at the University of Bristol. Briefly, a range of foods will be photographed and then presented to participants over a series of trials in a two‐alternative forced‐choice task. The ‘expected satiety’ value of the foods will be measured, along with an assessment of their anticipated healthiness, palatability and calorie content. For each participant, binary logistic regression will be used to estimate the relative weighting that is placed on each variable. By comparing models across groups of participants, it is possible to expose subtle differences in ‘food‐choice architecture’.

Independent of any direct effect on palatability, the energy density of food is likely to play an important role in food choice (Charbonnier *et al*. 2015). As snacks are often energy dense, a version of the choice‐architecture paradigm will be trialled which includes foods that differ in energy density and incorporates this variable in the modelling process. A potential concern is that modelling overall energy content masks subtle differences in the value that is placed on individual macronutrients. Evidence for macronutrient‐specific appetites (especially for protein) is mixed (Berthoud *et al*. 2012; Carreiro *et al*. 2016). Nevertheless, one possibility is that the values placed on calories from fat, carbohydrate and protein differ, and this may vary with age. The choice‐architecture paradigm could be ideally placed to identify evidence of this kind. Therefore, a feasibility study will be conducted to explore the modelling of responses to individual macronutrients, together with the role played by associated sensory characteristics (fatty taste, savouriness and sweetness). The validity of evidence for differential responses will be tested by exploring whether similar models are generated using different sets of foods and whether the value that is ascribed to different macronutrients remains constant, even in people who follow different diets (*e.g*. when meat‐free snacks are evaluated by vegetarians).

In a second phase, a larger study will be conducted that will be powered to quantify determinants of food choice in groups of mid‐life (40–54 years), younger old (55–69 years) and older old (70+ years) healthy participants. In so doing, the objective is to characterise the developmental trajectory of food‐choice strategies in healthy ageing, with particular focus on the relative role of energy density and protein as independent drivers of choice. In all cases, measures of trait dietary behaviours will be obtained, together with anthropometric data including an assessment of body mass index and body composition.

## Objective 3: To develop and disseminate a set of design rules for formulation of palatable, higher‐protein foods, for an ageing population

Building on the outcomes of objectives 1 and 2, academics and industry stakeholder partners will develop a design brief for formulation of palatable, higher‐protein foods, for older adults. This will draw upon existing literature on optimal sources of protein, taking into account amino composition, bioavailability, palatability, sustainability and acceptability, and be guided by the industry partners with consideration to technical implications of using new ingredients, such as cost, palatability and impact on manufacturing the final product. For example, protein as a food ingredient can be simply considered as existing in two basic forms: (1) a whole food ingredient with high protein content, for example a nut (almond), seed (pumpkin seed), grain (rice), legume (pea), algae or fungi; and (2) an extruded protein fraction, available as a concentrate, isolate or flour. Protein fractions can further be processed into textured vegetable proteins (TVPs) by force extrusion, steam injection, jet cooking or acid‐salt coagulation. The result is a (food) product with a defined texture, appearance and functionality. These physico‐chemical properties will determine the functionality during manufacture (ease of incorporation into the food matrix), palatability of the product (flavour, texture, aroma, appearance, mouthfeel) and ultimately the final production cost. Nutritional quality can also dictate a protein's functionality, as protein content, amino acid profile, digestibility and bioavailability become increasing essential constraints in product design briefs. We will thus highlight the type, amount and format of products with the greatest ability to influence protein intake, with consideration given to the sustainability of protein (Johnstone 2012).

As protein is a nutrient, and people eat food(s) not nutrients, the design brief will address not only protein type, quality and per meal dose (g), but also the timing of ingestion, as this can influence the consumers’ experience of palatability and the food's functional ability to influence health outcomes in an ageing population.

Relevant factors linked to age, such as chronic illness and malnutrition, will also be considered in terms of protein requirements (Mercer *et al*. 2015). For example, there is now substantial evidence that protein intakes above the current RNI can help to promote healthy ageing, appetite regulation, weight management and goals aligned with athletic performance (Phillips *et al*. 2016). The European Society for Clinical Nutrition and Metabolism Expert Group (ESPEN) makes the following recommendations for older populations taking into account protein intake, illness and activity levels:

1. for healthy older people, the diet should provide at least 1.0–1.2 g protein/kg bodyweight/day;
2. for older people who are malnourished or at risk of malnutrition because they have acute or chronic illness, the diet should provide 1.2–1.5 g protein/kg bodyweight/day, with even higher intake for individuals with severe illness or injury; and
3. daily physical activity or exercise (resistance training, aerobic exercise) should be undertaken by all older people, for as long as possible (Deutz *et al*. 2014).

## Objective 4: Formulate and trial at least one exemplar product based on the design rules

An exemplar/model product(s) for consumer assessment will be developed, based on the design brief (Objective 3). The production of the exemplar products will take place at Campden BRI (Gloucestershire, UK) and be informed by the technical and commercial expertise of the stakeholder industry partners. Ingredients and process technologies will be recommended by the industry stakeholders to increase the acceptability of the product's flavour and mouthfeel. Industry stakeholders will begin to develop model systems and processes to address any technological issues identified. Once developed, Campden BRI will supply exemplar products for consumer testing at the Universities of Aberdeen, Bristol, Newcastle and Sheffield, where participants will evaluate each exemplar product alongside a comparable, commercially available product. Participants will be asked to rate a range of the products’ characteristics including palatability, mouthfeel, acceptability and expected satiety. This sensory and consumer assessment represents a formal test of the design rules. The test will either validate these rules or provide a formal feedback loop to identify areas for improvement.

## Objective 5: Dissemination

Findings from the *Protein for Life project* will be disseminated via both scientific and lay communications (articles, presentations), along with a toolkit for stakeholder engagement. The toolkit will include infographics, short video summaries, press release statements and PowerPoint slides for uptake by various stakeholders (*e.g*. NHS, food industry, food retailers, the public and academics).

## Conclusions

The *Protein for Life* project will use a multidisciplinary approach to address a public health issue that is of major relevance to the food industry and provide a unique insight into the barriers to and opportunities for increasing protein intake in older adults. Industry will be provided with evidence‐based information for new product development or reformulation of existing products to help older adults improve health through increased protein intake.

## Author contributions

Emma Stevenson, Jeff Brunstrom, Alexandra Johnstone, Bernard Corfe, Elizabeth Williams, Mark Green and Anthony Watson all contributed equally to the writing and preparation of the manuscript.

*Protein for Life* academic team: Professor Emma Stevenson, Newcastle University (Principal Investigator); Professor Jeff Brunstrom, University of Bristol (Co‐Investigator); Professor Alexandra Johnstone, University of Aberdeen (Co‐Investigator); Dr Bernard Corfe, University of Sheffield (Co‐Investigator); Dr Elizabeth Williams, University of Sheffield (Co‐Investigator); Dr Mark Green, University of Liverpool (Co‐Investigator); Dr Anthony Watson, Newcastle University (Post‐doctoral researcher).

*Protein for Life* Industry Stakeholders: Campden BRI; Mondelez Int.; Nestle; Sainsbury's; Bradgate Bakery; Pladis; Premier Foods.

## Conflict of Interest

The authors declare no conflict of interest.
